# On the Structural Performance of Recycled Aggregate Concrete Columns with Glass Fiber-Reinforced Composite Bars and Hoops

**DOI:** 10.3390/polym13091508

**Published:** 2021-05-07

**Authors:** Ali Raza, Ahmad Rashedi, Umer Rafique, Nazia Hossain, Banjo Akinyemi, Jesuarockiam Naveen

**Affiliations:** 1 Department of Civil Engineering, University of Engineering and Technology, Taxila 47080, Pakistan; ali.raza@uettaxila.edu.pk; 2 College of Engineering, IT & Environment, Charles Darwin University, Casuarina 0810, Australia; 3 NUST Institute of Civil Engineering, National University of Science and Technology (NUST), Islamabad 44000, Pakistan; umerrafiq92@yahoo.com; 4 School of Engineering, RMIT University, Melbourne 3001, Australia; bristy808.nh@gmail.com; 5 Department of Agricultural and Biosystems Engineering, Landmark University, Omuaran 251101, Nigeria; akinyemi.banjo@lmu.edu.ng; 6 School of Mechanical Engineering, Vellore Institute of Technology, Vellore 632014, India; naveen.j@vit.ac.in

**Keywords:** recycled aggregate concrete, GFRP, geopolymer concrete, structure, strength, failure, material properties

## Abstract

Structural members comprising geopolymer recycled aggregate concrete (RAC) reinforced with glass fiber-reinforced polymer (GFRP) bars have not been investigated appropriately for axial compressive loading cases. The present study addresses this knowledge gap by evaluating the structural efficiency of GFRP-reinforced geopolymer recycled aggregate concrete (GGRAC)-based members subjected to axial compressive loading. A total of nine compressive members (250 mm in cross-section and 1150 mm in height) were constructed to examine the effect of the number of longitudinal GFRP bars and the vertical spacing of transverse GFRP hoops/ties. The experimental results portrayed that the ductility of GGRAC compressive members improved with the reduction in the pitch of GFRP hoops. The axial load-carrying capacity (LCC) of GGRAC compressive members increased by increasing the number of GFRP bars up to eight (corresponding to a reinforcement ratio of 2.11%) while it decreased by using ten longitudinal GFRP bars (corresponding to a reinforcement ratio of 2.65%). Additionally, an empirical model was suggested to predict the axial LCC of GGRAC compressive members based on a large amount of experimental data of similar members. The experimental results and related theoretical predictions substantially prove the applicability and accuracy of the proposed model. The proposed column represents a feasible structural member in terms of material availability and environmental sustainability.

## 1. Introduction

The quantity of construction and demolition (C&D) waste, as a result of the demolition of old infrastructures, is increasing around the world due to an increase in world population, extensive urbanization, and rapid development of developing countries. Therefore, it is necessary to utilize this C&D waste in a proper way to develop a sustainable environment. Recycled aggregate concrete (RAC) lessens carbon dioxide emission, land required for the C&D waste, and aggregate transportation distance by meeting the desire for environmentally friendly, low-carbon, and sustainable production [1,2,3,4,5,6]. Even though RAC has many deficiencies similar to normal concrete, such as high porosity and less compressive strength, there is still one significant benefit of RAC from a ductility point of view [7]. Carbon dioxide is the byproduct of the production of Portland cement. To diminish the carbon footprint of concrete construction, a green concrete named ‘geopolymer concrete’ (GPC) consisting of recycled coarse aggregates (RCA) was utilized in the present study. GPC concrete utilizes a binder consisting of alumino-silicate polymers produced from alkali activators such as blast furnace slag, red mud, silica fume, and fly ash. Although the mechanical and durability performances of RAC and GPC have been explored by various researchers, the usage of fiber-reinforced polymer (FRP) bars in GPC-RAC still has not been investigated up to the requirement. Glass fiber-reinforced polymer (GFRP) bars have superior features over steel bars such as lower weight, higher corrosion resistance, lower electromagnetic interference, lower thermal conductivity, and higher tensile strength [8,9,10,11,12,13]. The use of FRP bars in aggressive environments lessens repair costs, increases serviceability, and prolongs the serviceable life of structures [9,14,15,16,17,18,19,20,21,22,23,24].

Currently, the applications of GFRP reinforcement in concrete structures have drawn strong interest from researchers. A comparative investigation on the axial compression performance of GFRP and steel-reinforced compressive members portrayed that the load-carrying capacity (LCC) of GFRP-RC compressive columns was 7% lower than that of steel-reinforced compressive members [25]. The GFRP-RC compressive members with adequate lateral confinement bear peak loads identical to or higher than steel-reinforced compressive members which firmly demonstrates the applications of GFRP bars in compressive members [26,27]. The tests of GFRP-RC compressive members indicated that a volumetric ratio of 0.7% for the lateral GFRP reinforcement showed a buckling of GFRP bars during failure and volumetric ratios of 1.5% and 2.7% for the lateral GFRP reinforcement showed a rupture of transverse ties and the crushing of the core during the failure process [28]. The tests of circular GFRP-RC compressive members depicted that the moment and axial LCC of columns were less than their steel-reinforced equivalents [29]. Xiong et al. [30] explored the axial compressive behavior of FRP-wrapped steel-reinforced RAC (FCSRC) compressive members. They concluded that the FCSRC compressive members presented similar compressive performance compared with natural aggregate concrete-based compressive members, except for the axial compressive strength that slightly decreased with the replacement of natural coarse aggregates (NCAs) with RCA.

Hadi et al. [31] explored the structural response of GPC compressive members reinforced and confined with basalt fiber-reinforced polymers. They concluded that the axial compressive strengths of steel-reinforced GPC compressive members were 27% to 34% higher while their ductility was 16% to 27% less than that of steel-reinforced ordinary Portland cement concrete compressive members. Furthermore, the GPC compressive members reinforced and confined with BFRP bars and tubes presented 5% to 19% lower axial compressive strengths and 4% to 7% higher ductility than the OPC compressive members reinforced and confined with BFRP bars and tubes. Danda et al. [32] studied the effect of molarity of NaOH on the axial compressive behavior of reinforced GPC compressive members using GGBS and determined that the increase in the molarity of NaOH resulted in the improvement of LCC and reduction in the axial deflection of compressive members. During the manufacturing of GPC, the activation process results in increased energy consumption and the production of greenhouse gases that present a safety risk. In addition, the fabrication of GPC is significantly influenced by the curing temperature and duration [33,34]. Saranya et al. [35] explored the behavior of steel fiber-reinforced dolomite-GGBS GPC short compressive members subjected to axial compressive loading and found that these compressive members portrayed higher axial compressive strength than steel fiber-reinforced OPC compressive members. Additionally, dolomite-GGBS GPC compression members without ductile detailing to provide adequate toughness and ductility presented a similar behavior compared with that of OPC compression members with ductile detailing, giving a reduced cost to strength ratio. Maranan et al. [36] studied the structural response of GFRP-reinforced GPC compressive members, concluding that these compressive members showed superior compressive performance compared with their OPC counterparts. The spiral-confined GPC compressive members portrayed higher confinement effectiveness and ductility compared with hoop-confined GPC compressive members.

An extensive literature review reveals that the axial behavior of GFRP-reinforced geopolymer recycled aggregate concrete (GGRAC) compressive members has not been investigated. This work aims to inspect the structural behavior of GGRAC compressive members by carrying out experiments under axial compressive loading and to propose a novel empirical equation for the axial strength of GGRAC columns based on a large experimental database, which is the novelty of the present work. The influences of GFRP bars and pitch of lateral GFRP hoops on the axial LCC, axial deflection, failure modes, and cracking behavior of GGRAC compressive members were investigated. Additionally, a mathematical model was suggested for estimating the axial compressive strength of GGRAC compressive members. The proposed column is a feasible structural member in terms of cost, material availability, and environmental sustainability factor. The outcome of this study can be supportive of structural engineers while analyzing and designing such green concrete compressive members.

## 2. Materials and Methodology

### 2.1. Materials

#### 2.1.1. Geopolymer Recycled Aggregate Concrete

**Figure 1:**
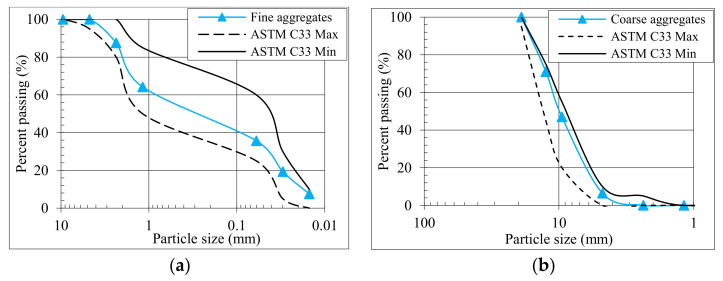
Granulometric analysis of (**a**) fine aggregates (**b**) recycled coarse aggregates.

In the preparation of GRAC, the 100% replacement of natural aggregates with recycled aggregates was done. To obtain the RCAs for the construction of compressive members, the tested cylinders with a compressive strength of 30–45 MPa were crushed at the age of one year. Table 1 reports different features of recycled aggregates. Ten millimeters was the maximum size of recycled aggregates. Lawrancepur sand, locally available in the region, was utilized as fine aggregates, having an apparent density of 2632 kg/m^3^ and a fineness modulus of 2.25. Figure 1 presents the granular analysis of RAC and sand utilized in the present work. The data of Figure 1 were obtained after performing the sieve analysis of aggregates in the laboratory. Superplasticizer, called Sika ViscoCrete^®^-3425, was utilized to ensure satisfactory workability of the GPC mix. The mix design of GPC (reported in Table 2) with a density of 2,400 kg/m^3^ was obtained using a hit-and-trial method by knowing the water absorption of recycled aggregates. Two commercially available waste materials, i.e., 45% class F fly ash and 55% ground granulated blast-furnace slag (GGBS), were utilized as binders in GRAC. A mixture of NaOH (14M molarity) and Na_2_SiO_3_ was utilized as an activator in a mass ratio of 1:2.5. According to ASTM C143 [37], the slump test showed a slump value of 125 mm of fresh GRAC. The setting time of GPC following ASTM C807-13 [38] was 90 min. Six concrete cylinders (150 mm × 300 mm) showed a compressive strength of 34.5 MPa when tested from the same mix at 28 days of age with a deviation of 3 MPa.

#### 2.1.2. GFRP Bars

**Figure 2:**
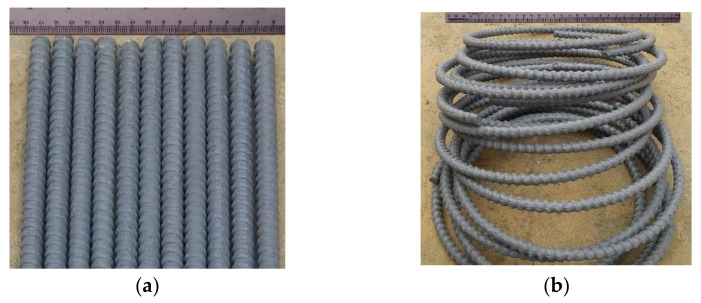
GFRP reinforcement: (**a**) longitudinal bars (**b**) hoops.

The compressive test members were made using 9.5 mm diameter GFRP hoops and 12.7 mm diameter GFRP bars as a lateral and longitudinal reinforcement, correspondingly. The splice length of GFRP hoops was 65 mm. The GFRP bars were obtained from SupAnchor^®^. They were fabricated with an 80% fibrous material and impregnated with thermosetting vinyl ester and additives. The tensile properties of the GFRP bars were measured by performing the B.2 test suggested by ACI 440.3R [39] and CAN/CSAS807-10 [40]. The physical and mechanical features of GFRP bars are presented in Table 3. The GFRP longitudinal bars and GFRP hoops utilized in this investigation are presented in Figure 2.

### 2.2. Specimen Details and Fabrication

**Figure 3:**
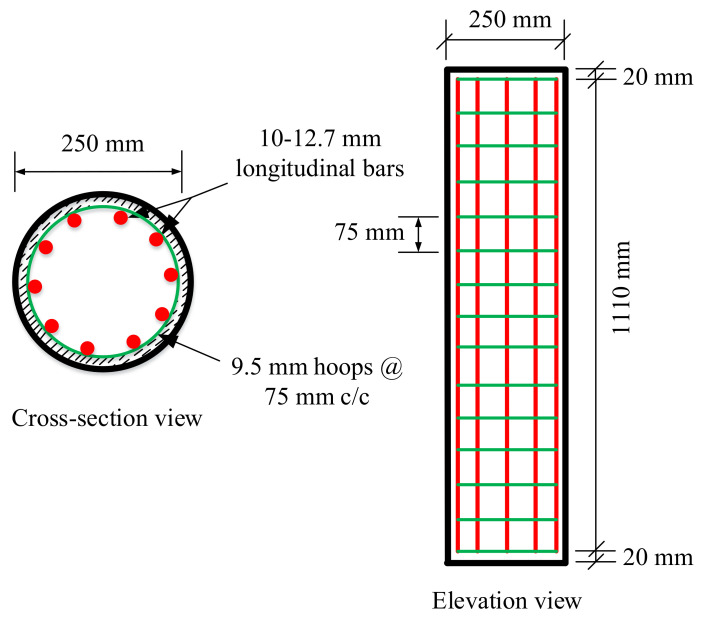
Schematic diagram of a specimen with 75 mm pitch of GFRP hoops.

In the current study, a total of nine circular GGRAC compressive members were fabricated and tested to examine the effect of different GFRP bars and vertical pitch of GFRP ties on the structural performance of compressive members. The diameter of the compressive members was 250 mm and the height of compressive members was 1150 mm, making them small enough to fit in the available compression machine and large enough to be considered as full-size compressive members. Three groups of compressive members were manufactured. Each group consisted of three compressive members with six, eight, and ten GFRP bars of 12.7 mm diameter, respectively. In the first, second, and third compressive members of each group, the spacing of GFRP hoops (9.5 mm thick) was 75 mm, 150 mm, and 250 mm, respectively. The longitudinal GFRP reinforcement ratios in the first, second, and third groups were 1.57%, 2.11%, and 2.65%, respectively. These low reinforcement ratios could be preferred in low seismicity zones [22,23]. The slenderness ratios of all specimens were kept at 4.6, defining them as short columns. Furthermore, the diameters of reinforcing bars were chosen by keeping the size of compressive members and axial compression capacity of the laboratory instrument in mind. The volumetric ratios of transverse GFRP hoops were 1.42%, 0.71%, and 0.50% for the first, second, and third compressive members of each group, respectively. The spacing of transverse GFRP hoops was adjusted so that the elastic buckling of GFRP bars could be ensured [36]. The concrete cover was kept at 20 mm for all test compressive members. The geometric and cross-sectional specifications of a specimen with 10 GFRP bars and 75 mm transverse GFRP hoop spacing are presented in Figure 3.

The compressive members were labeled using two digits and five letters. The first letter “G” indicates GFRP reinforcement, the second letter “G” indicates geopolymer concrete, “RAC” represents recycled aggregate concrete, the last digit designates the number of longitudinal GFRP bars, and the right-hand side digit represents the spacing of transverse GFRP hoops. For example, GGRAC8-250 is a GGRAC column that contains six GFRP longitudinal bars and GFRP lateral hoops at a clear spacing of 250 mm. Table 4 reports the geometric and testing particulars of the compressive members. Five mm thick PVC pipes with an inner diameter of 250 mm were utilized as a formwork for the compressive members. For providing the concrete cover, spacers were utilized. The mixing of GPC was done at 20 revolutions/min in a mechanical mixer having a capacity of 0.15 m^3^. Twenty hours before the preparation of GPC, a solution of water and NaOH was made. About 30 min before the mixing of GPC, Na_2_SiO_3_ was mixed with the solution of water and NaOH. First of all, the dry constituents were mixed thoroughly, and then the activation solution was mixed for 2 min. After that, the water and superplasticizer were poured and mixed until a homogenous mix was obtained. The reinforcement cages were reinforced with GFRP bars and concrete was placed along with a continuous vibration to remove the air voids and bubbles produced in the concrete. The curing of the GGRAC compressive members was performed at normal temperature by casing them with polyethylene sheets to prevent moisture loss.

### 2.3. Testing and Instrumentation

**Figure 4:**
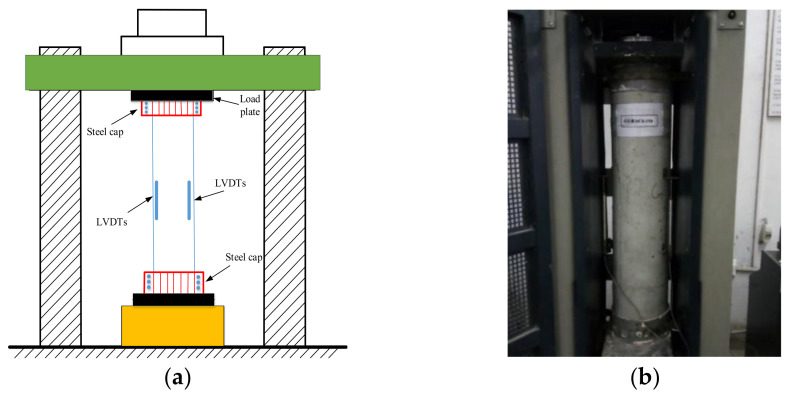
Testing of compressive members in the present study: (**a**) schematic diagram (**b**) experimental setup.

The testing of GGRAC compressive members was carried out in the Controls MCC8 compression equipment having a compressive capacity of 5000 kN. The testing procedure was according to ref. [20,22,23]. For the application of uniform load on the surface of compressive members, plaster of Paris was utilized for smoothing and flattening the surface. Furthermore, steel collars (10 mm thick and 100 mm wide) were applied to both ends of the specimens for the end crushing, as presented in Figure 4. First, a preload of 100 kN was applied to the compressive members using a load control technique to remove any misalignment. The loading rate was 50 kN/min. The compressive members were unloaded at the same speed and then loaded using a displacement control technique at a compression rate of 0.03 mm/s. Three different linear variable differential transformers (LVDTs) with 300 mm gauge length were attached at 120° apart to the specimens to measure the axial compressive deflections. The readings of LCC and axial deflection were noted utilizing a data logger connected to the testing machine. A video recorder was employed to detect the failure modes and cracking behavior of the compressive members.

## 3. Results and Discussion

### 3.1. Load–Deflection Behavior

The experimental results for the peak LCC, the deflections at the peak LCC, and the ultimate deflections of GGRAC compressive members were reported in Table 5. The maximum LCC was given by the specimen GGRAC8-75, having a value of 1777.3 kN that was 7% higher than the specimen GGRAC6-75. The highest ductility index was also presented by the specimen GGRAC8-75 due to its higher energy absorption and higher stiffness in the post-peak loading stage. Column GGRAC8-75 initially had a linear rising slope that was up to an axial load of 1758.6 kN and axial deformation of 2.83 mm. After that, a slight nonlinear climbing part seemed to be due to crack propagation, ending with a peak axial LCC of 1777.3 kN at 3.25 mm.

The specimen GGRAC6-75 presented a linear elastic response up to 1446.8 kN at an axial compression of 2.02 mm. After that, it showed less improvement in the axial load due to the development and propagation of vertical hairline cracks in the middle portion of the specimen. Finally, reaching an axial compression of 1652.8 kN at an axial deflection of 3.24 mm, it continued to deform, causing a reduction in the stiffness and strength of the specimen. The specimen GGRAC10-150 presented a linear elastic response up to a peak value of 1587.4 kN at an axial deflection of 3.56 mm. After that, it showed a sudden decrease in the axial load due to the development and propagation of vertical hairline cracks in the middle portion of the specimen. Finally, reaching an axial compression of 685.3 kN at an axial deflection of 4.79 mm, the specimen collapsed. The compressive members with eight GFRP bars showed a higher stiffness and less reduction in the axial loading capacity in the post-peak failure behavior.

### 3.2. Failure Process

**Figure 5:**
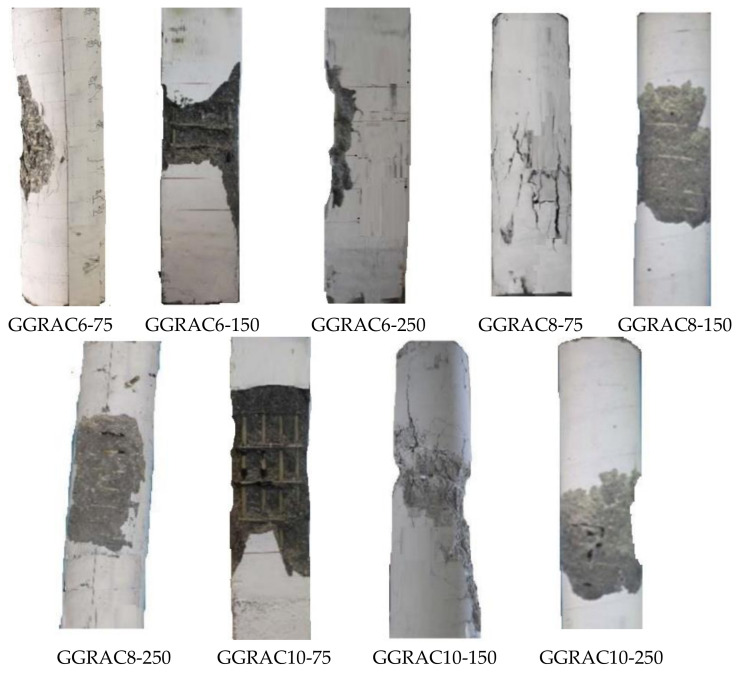
Crack initiation of GGRAC compressive members.

The failure of all the GGRAC compressive members occurred in the middle region due to the buckling of the main GFRP bars and the breaking of lateral GFRP ties. After the application of the axial compressive load, the compressive members behaved elastically up to about 85% of the peak load. At this point, the confining influence given by the transverse GFRP ties was not triggered. When the compressive load was increased more, the GRAC initiated cracking, having a vertical hairline crack and a low sound in the compressive members. After gradually increasing the load up to the peak value, the cracks extended along the length of compressive members, and the width of cracks was amplified. The cracking of the concrete cover started and GFRP ties were activated to deliver lateral confinement to the core. Finally, the test compressive members consecutively showed the fracture of GFRP hoops at about 65% of the maximum amount in the post-peak behavior; breakage of main GFRP bars; and finally, the crushing of GRAC core restricted with lateral GFRP hoops. Figure 5 represents the experimental crack initiation and propagation.

### 3.3. Ductility

**Figure 6:**
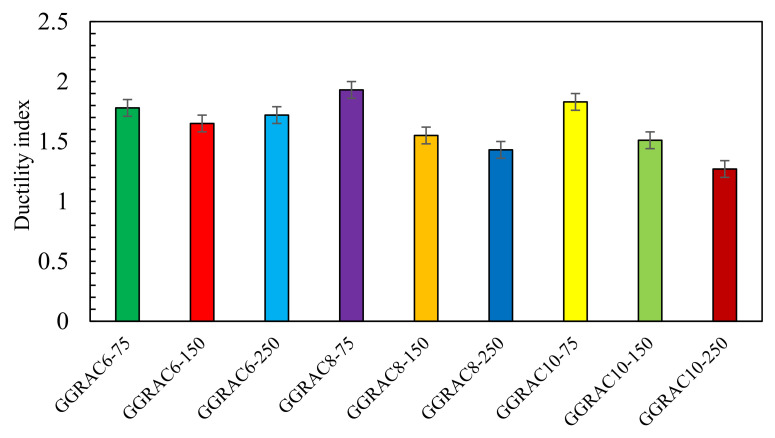
Ductility indices for various GGRAC compressive members.

Ductility is the capacity of the structural elements to absorb energy. For GGRAC compressive members, the ductility index was calculated as the ratio of the area under the load–deflection curve up to 85% of peak load in the post-collapse behavior to the area under the load–deflection behavior up to 75% of peak load in the pre-peak loading, as evidenced by the previous studies [41,42,43]. Figure 6 presents the ductility indices for all GGRAC compressive members. The compressive members with a smaller spacing of stirrups showed higher ductility indices. The compressive members GGRAC6-75, GGRAC8-75, and GGRAC10-75 showed ductility indices of 1.78, 1.93, and 1.83, respectively.

The highest energy was absorbed by the specimen GGRAC8-75 due to good confinement. The spacing of transverse GFRP ties significantly affected the ductility index, while the longitudinal reinforcement ratio did not show any specific effect on this parameter. For example, GGRAC6-75 showed an index of 1.78 while GGRAC6-250 showed an index of 1.65. Conversely, GGRAC6-75 showed a ductility index of 1.78 and GGRAC10-75 showed an index of 1.83. The average ductility indices were 1.72, 1.64, and 1.54 for the GGRAC compressive members having the stirrups spaced at 75 mm, 150 mm, and 250 mm, respectively. The compressive members with high reinforcement ratios presented lower values of ductility indices due to their brittle behavior, allowing low energy absorption. The improvement in the ductility of the GGRAC compressive members with smaller stirrup spacing may be attributed to well-restrained GFRP bars and the operative lateral confinement of the GRAC core to engage more energy [43].

### 3.4. Effect of Longitudinal Reinforcement Ratio

**Figure 7:**
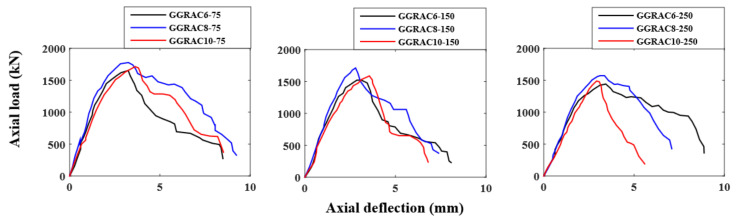
Influence of reinforcement ratio on the load–deflection response of GGRAC compressive members.

Figure 7 portrays the influence of longitudinal reinforcement ratio on the complete load–deflection performance of GGRAC compressive members. No significant improvements in the axial LCC of compressive members were observed by increasing the longitudinal reinforcement ratio except making the compressive members more brittle. By enhancing the number of bars from six (1.57% reinforcement ratio) to eight bars (2.11% reinforcement ratio) and ten bars (reinforcement ratio of 2.65%), the improvements in the axial LCC of GGRAC compressive members with 75 mm spacing of hoops were 7% and 3.5%. By increasing the reinforcement ratio from 1.57% to 2.11% and 2.65%, the improvements in the axial LCC of GGRAC compressive members with 150 mm stirrup spacing were 11.2% and 4.2%. Similarly, by increasing the reinforcement ratio from 1.57% to 2.11% and 2.65%, the improvements in the axial LCC of GGRAC compressive members with 250 mm stirrup spacing were 8.3% and 3.2%. Therefore, high longitudinal reinforcement ratios did not present a significant increase in axial capacity. However, the compressive members with a larger number of GFRP bars presented a higher axial stiffness response due to a larger effectiveness ratio of axial stiffness of GFRP bars to the effective GRAC cross-sectional area.

### 3.5. Effect of Stirrup Spacing

**Figure 8:**
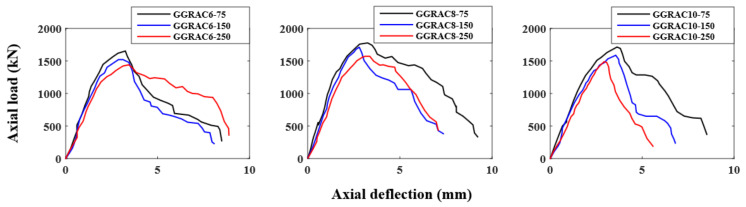
Influence of pitch of GFRP hoops on the load–deflection behavior of GGRAC compressive members.

Figure 8 depicts the influence of the pitch of GFRP hoops on the load–deflection performance of GGRAC specimens. The decrease in the pitch of GFRP hoops provided an enhancement in the LCC of GGRAC compressive members. By reducing the GFRP tie spacing from 150 mm to 75 mm, a rise of 7.99% was observed for GGRAC compressive members with six longitudinal bars. When the pitch of GFRP hoops was decreased from 250 mm to 150 mm, a rise of 12.82% was detected for GGRAC compressive members with six longitudinal bars. By reducing the GFRP tie spacing from 150 mm to 75 mm, a rise of 3.65% was detected for GGRAC compressive members with eight longitudinal bars. When the pitch of GFRP hoops was decreased from 250 mm to 150 mm, a rise of 11.60% was detected for GGRAC compressive members with eight longitudinal bars. Similarly, by reducing the GFRP tie spacing from 150 mm to 75 mm, a rise of 7.37% was detected for GGRAC compressive members with ten longitudinal bars. When the pitch of GFRP hoops was decreased from 250 mm to 150 mm, a percentage rise of 13.16% was observed for GGRAC compressive members with ten longitudinal bars. The increase in the LCC of the GGRAC compressive members with the decrease in the stirrup spacing is because of the well-restrained reinforcement and efficient confinement of the GRAC core [43].

## 4. Theoretical Calculations

### 4.1. Experimental Database

A huge database of 250 FRP-RC compressive members was established from previous works to propose a new empirical model that was validated using the experimental results of GGRAC compressive members tested in the present investigation. Longitudinal reinforcement provided in all the samples was by FRP bars but the transverse confinement was provided using FRP spirals, FRP hoops, or steel hoops. The geometry of 116 compressive members was square/rectangular, while that of 134 compressive members was circular. The transverse confinement in 31 compressive members was provided using steel hoops, in 101 compressive members it was provided using GFRP hoops, in 8 compressive members it was provided using CFRP spirals, and in 110 compressive members it was provided using GFRP spirals. The developed database consisted of the strength of FRP bars ($f_{u}$), the breadth of columns (B), the width of columns (D), the height of columns (H), the compressive strength of concrete ($f_{c}^{’}$), the elastic modulus of FRP bars ($E_{f}$), peak strain of FRP bars ($\varepsilon_{u}$), transverse reinforcing ratio ($\rho_{t}$), reinforcing ratio of longitudinal FRP bars ($\rho_{l}$), and, lastly, LCC of FRP-RC compressive members ($P_{n})$. Table 6 presents the statistical details of the database and various parameters of the experimental database are provided in the Supplementary Materials. 

### 4.2. Assessment of Previous Equations

The database developed for GFRP-RC compressive members was employed to assess the different empirical models that were taken from the previous research to acquire a general form of the recently proposed model for axial LCC [25,28,44,45,46,47,48,49,50,51,52,53]. These models were assessed by using some statistical indices, coefficient of determination (*R*^2^), the mean absolute error (*MAE*), and the root mean squared error (*RMSE*), as reported by Equations (1)–(3), respectively. Of all the statistical parameters, *R*^2^ is the most crucial and the most appropriate. Therefore, the experimental results were utilized for performing the comparative study using this parameter.

(1) $$R^{2}={\left(\frac{n\left(\sum_{i=1}^{n}x_{i}y_{i}\right)-\left(\sum_{i=1}^{n}x_{i}\right)\left(\sum_{i=1}^{n}y_{i}\right)}{\sqrt{\left[n\sum_{i=1}^{n}x_{i}^{2}-{\left(\sum_{i=1}^{n}x_{i}\right)}^{2}\right]\left[n\sum_{i=1}^{n}y_{i}^{2}-{\left(\sum_{i=1}^{n}y_{i}\right)}^{2}\right]}}\right)}^{2}$$

(2) $$MAE=\frac{1}{n}\overset{n}{\underset{i=1}{\sum }}\left|x_{1}-y_{1}\right|$$

(3) $$RMSE=\sqrt{\frac{1}{n}\overset{n}{\underset{i=1}{\sum }}{\left(x_{1}-y_{1}\right)}^{2}}$$

**Figure 9:**
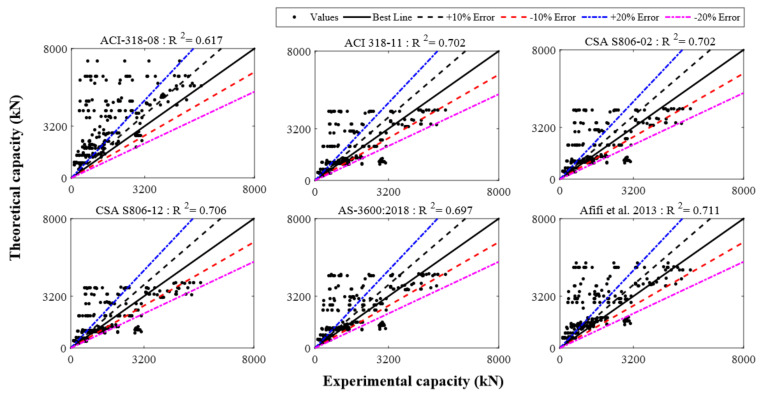

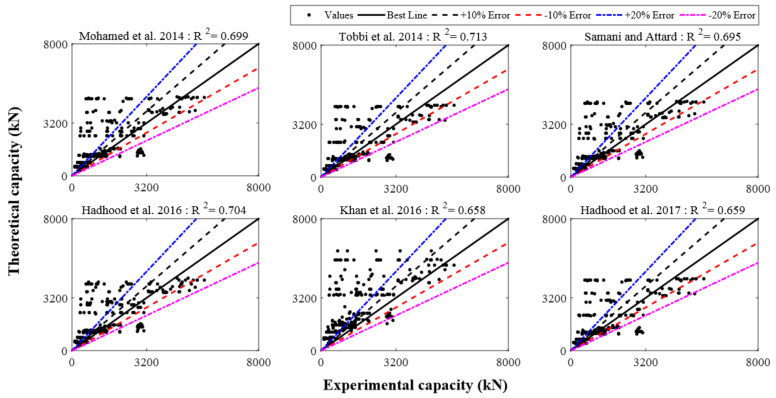
The behavior of existing strength models over the database.

Here, *n* shows the total number of the sample points, *x* is the LCC of GFRP-RC compressive members attained from experiments, and *y* is the value of LCC of GFRP-RC compressive members given by the mathematical models. Figure 9 shows the assessment of all empirical models.

### 4.3. Confinement Effect

The effect of lateral confinement provided by the GFRP ties led to improving the ductility and strength of GFRP-RC compressive members [54]. After the application of compressive load, this confining influence enhances by limiting the lateral dilation of concrete because of the lateral pressure when the specimen crosses the peak LCC. In the previous studies, the LCC of GFRP-RC compressive members was estimated without bearing in mind the confinement influence of GFRP ties. By ignoring the influence of GFRP imprisonment on the LCC of compressive members, the estimations are underestimated [55]. Therefore, the models proposed by Afifi et al. [54] were utilized in the present work for the calculations of the stress and strain of FRP-confined concrete (Equations (4) and (5)).

(4) $$\frac{f_{cc}^{’}}{f_{co}^{’}}=1.0+4.547{\left(\frac{f_{le}}{f_{co}^{’}}\right)}^{0.723}$$

(5) $$\frac{\varepsilon_{cc}^{’}}{\varepsilon_{co}^{’}}=1.0+\left(\frac{0.024}{\varepsilon co’}\right){\left(\frac{f_{le}}{f_{co}^{’}}\right)}^{0.907}$$

The transverse confinement stress (*f_l_*) due to GFRP hoops can be reported by Equation (6) [56].

(6) $$f_{l}=k_{e}\frac{2f_{fb}A_{tf}}{sd_{s}}$$

where $k_{e}=A_{e}/A_{c}$ is the confinement effectiveness coefficient, $A_{c}$ is the concrete core area, $A_{e}$ is the effectively confined core area, $f_{fb}$ is the bending strength of GFRP ties calculated as 0.004$E_{ft}$ [44], $A_{tf}$ is the area of GFRP hoops, $d_{s}$ is the diameter of concrete, and s is the vertical distance between GFRP hoops.

### 4.4. Proposed Equation for Axial Load-Carrying Capacity

The model given by Tobbi et al. [49] portrayed the highest accuracy with *R*^2^ = 0.713. When *R^2^* has a value of one, it shows that there is a perfect correlation of experimental measurements with the predictions for LCC. Thus, the general form of the newly recommended model was taken to be like that of Tobbi et al. [49]. On the contrary, the role played by FRP bars to affect the axial LCC of GFRP-RC compressive members was taken into account because of the elastic modulus and the area of FRP bars with a reducing factor. Equation (7) shows the general form of the currently recommended model. Although considering the fractal model concept in the modeling can give precise predictions [57], the fractal model concept of FRP bars (initial geometric imperfection and curvy geometrical figure) in the current investigation has been ignored to make the suggested model simple to avoid complexity of the model for the practical applications.

(7) $$P_{n}=\alpha_{1}\left(A_{g}-A_{FRP}\right)f_{cc}^{’}+\varepsilon_{cc}E_{FRP}A_{FRP}$$

In this model, $\alpha_{1}$ is the constant known as the reduction coefficient for the loading strength of GFRP-RC compressive members owing to a concrete core confined with FRPs, $A_{g}$ represents the cross-sectional area of the column, $A_{FRP}$ shows the cross-sectional area of FRP longitudinal bars, and $E_{FRP}$ shows the elastic modulus of FRP longitudinal bars. The curve fitting technique was utilized to determine the value for this constant. The error functions were minimized as much as possible to attain the most suitable fit. The relationship for $\alpha_{1}$ can be defined as $\alpha_{1}=0.85-\beta f_{c}^{’}$ where β is a constant. To get the most suitable fit to the experimental database, the curve fitting method was employed which gave the value of constant β as 0.0029. The model proposed for axial LCC of the GFRP-reinforced compressive members by considering the lateral confinement efficiency given by the GFRP ties can be reported as expressed by Equation (8):

(8) $$P_{n}=\left(0.85-0.0029f_{c}^{’}\right)\left(A_{g}-A_{FRP}\right)f_{cc}^{’}+\varepsilon_{cc}E_{FRP}A_{FRP}$$

**Figure 10:**
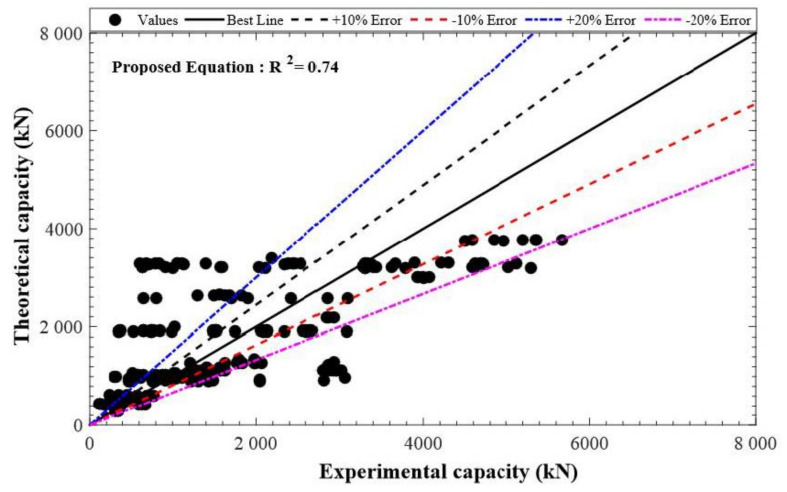
Performance of the newly suggested model.

The reducing factor should be higher than 0.794, i.e., $\alpha_{1}=0.85-0.0029f_{c}^{’}\geq 0.79$. Apart from some limitations, the newly suggested model was more precise than all of the formerly existing models. The range of parameter $f_{c}^{’}$ should range between 20 MPa and 70 MPa, the parameter $f_{FRP}$ should range between 406 MPA and 1680 MPa, while that of the ultimate strain of bars should range between 0.97 and 2.42%. The suggested equation (Equation (8)) functioned well when *R*^2^ = 0.74, as reported in Figure 10.

**Figure 11:**
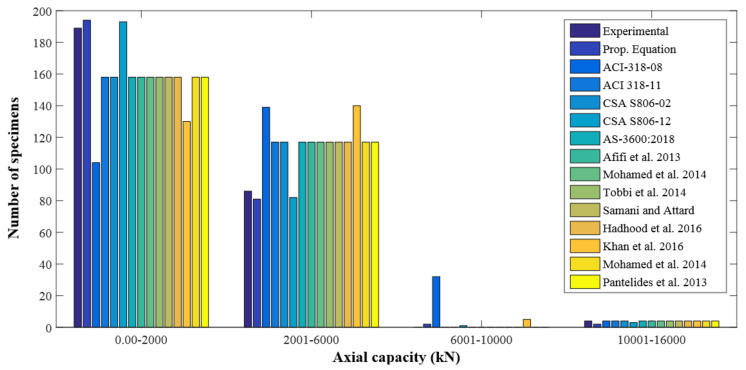
Distribution of axial strength of GFRP-RC compressive members.

**Figure 12:**
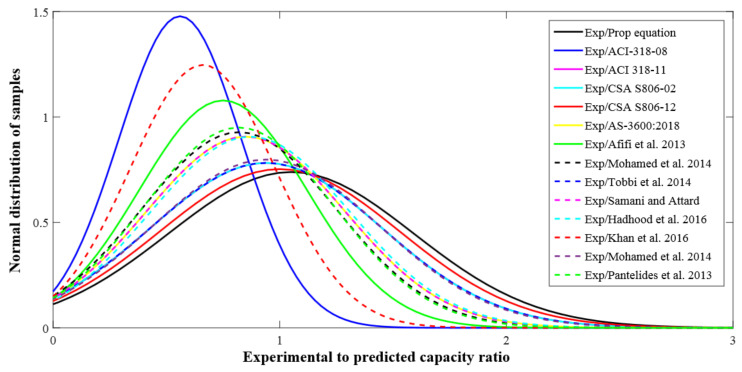
Normal distribution of experimental to predicted strength of GFRP-RC compressive members.

The distribution for the previous predictions, as well as the testing measurements for the axial strength of GFRP-RC compressive members, is reported in Figure 11. The database constructed had a total of 185 different test values for axial LCC ranging from 0–2000 kN. In this range, 165 values were provided by the proposed equation. Similarly, there were 86 experimental and 81 estimated values in the 2001–6000 kN range, 0 experimental and 2 estimated values in the 6001–10,000 kN range, and 4 experimental and 2 predicted values in the 10,001–16,000 kN range. These values go on to show that the equation put forward did a good job of describing the axial LCC of GFRP-RC compressive members. Figure 12 shows the normally distributed experimental and estimated values for the axial strengths of GFRP-RC compressive members. The data of this graph were attained from the normalized estimates of different previous equations over the constructed database. With a variation of only 4% from unity, the suggested equation has done well for the normalized values of ratios of experimental axial LCC to estimated LCC; 41% was the peak deviation for ACI-318-08 [45]. The deviation may owe to the intention that the recommended model by ACI-318-08 [45] is for conventional steel reinforcement but this model is employed only for relative study. Additionally, the fractional deviations for the equations given by Khan et al. [52], Afifi et al. [25], and CSA S806-12 [44] were 29%, 20%, and 7%, respectively.

**Figure 13:**
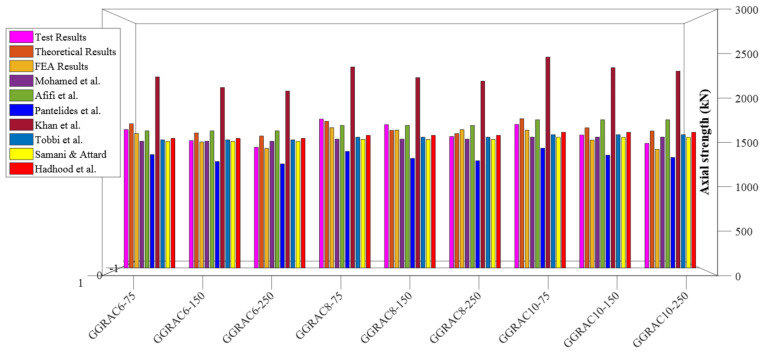
Comparison of estimates of different models for the LCC of GGRAC compressive members.

The comparative investigation depicted that the proposed model described the LCC of GRAC compressive members with high accuracy, as reported in Figure 13. The newly proposed mathematical model for the LCC of compressive members portrayed an average error of 5.18% for GGRAC compressive members. The previous models reported higher deviations for the axial LCC of GGRAC compressive members. Thus, the predictions of the proposed empirical models solidly substantiate its applicability and accuracy for satisfactorily capturing the LCC of GGRAC compressive members by bearing in mind the involvement of GFRP longitudinal and transverse bars. The proposed structural element is a feasible member in terms of cost, material availability, and environmental sustainability factor [58,59,60].

## 5. Conclusions

The present investigation aims to explore the structural behavior of GGRAC compressive members by carrying out experiments and theoretical assessments. The following key points can be obtained from this investigation:

1. All the GGRAC compressive members showed similar failure modes and processes. Commonly, the failure was detected in the middle portion of compressive members. The compressive members failed due to a fracture arising in the longitudinal bars and rupture in the GFRP ties.
2. The compressive members with a lesser spacing of GFRP hoops showed higher ductility indices because of the ability of the well-restrained longitudinal bars and efficient transverse confinement of the concrete to absorb greater energy.
3. The reduction in the pitch of GFRP hoops led to an increase in the LCC of GGRAC members. Reduction in the vertical pitch of GFRP hoops from 150 mm to 75 mm resulted in an improvement of 3.65% in the LCC of specimens. When the vertical spacing of GFRP hoops was reduced from 250 mm to 150 mm, a percentage reduction of 11.6% was noticed in the LCC of GGRAC specimens.
4. The increase in the quantity of longitudinal GFRP bars up to eight improved the axial LCC of GGRAC specimens while using ten longitudinal bars decreased the axial LCC of specimens.
5. The recommended mathematical model considered the axial involvement of the main GFRP bars and the confining phenomenon of lateral GFRP hoops and presented a discrepancy of only 5.18% from the experimental tests. These comparative assessments solidly authenticate the applicability of the suggested model for capturing the axial LCC of GGRAC compressive members. Consequently, the GFRP-reinforced geopolymer recycled aggregate concrete compressive members perform well in terms of axial LCC, failure modes, and ductility. The present study proposes a novel compressive member for sustainable and green concrete construction. Future work is recommended to examine the performance of GFRP reinforcement in various GPC members including beams and slabs.

## Figures and Tables

**Table 1:** Different features of recycled aggregates.

Feature	Value	Feature	Value
Water absorption	7.39%	Maximum size	10 mm
Apparent density	2632 kg/m^3^	Specific gravity	2.23
Bulk density	1295 kg/m^3^	Los Angeles abrasion	40.32%
10% fine value	135	Minimum size	4.75 mm

**Table 2:** Quantities of ingredients of GRAC.

Ingredient	Quantity	Ingredient	Quantity
RCA	1186 kg/m^3^	Fly ash	245 kg/m^3^
Sand	501 kg/m^3^	GGBS	163 kg/m^3^
Water	123 kg/m^3^	Superplasticizer	3.8 kg/m^3^
NaOH solution (14M)	39 kg/m^3^	Na_2_SiO_3_	105 kg/m^3^

**Table 3:** Different features of GFRP bars.

Bar Number	Diameter (mm)	Area (mm^2^)	Tensile Strength (MPa)	Elastic Moduli (MPa)	Ultimate Tensile Strain (%)
No. 3	9.5	70.8	765 ± 4	48,000 ± 500	2.12 ± 0.02
No. 4	12.7	126.6	830 ± 5	50,000 ± 500	2.03 ± 0.02

**Table 4:** Details of test compressive members.

Column Identifier	Longitudinal Reinforcement			Transverse Reinforcement		
	Nominal Diameter (mm)	No. of Bars	Reinforcement Ratio (%)	Nominal Diameter (mm)	Pitch (mm)	Volumetric Ratio (%)
GGRAC6-75	12.7 ± 0.3	6	1.57 ± 0.5	9.5 ± 0.2	75	1.42 ± 0.2
GGRAC6-150					150	0.71 ± 0.2
GGRAC6-250					250	0.50 ± 0.2
GGRAC8-75	12.7 ± 0.3	8	2.11 ± 0.5	9.5 ± 0.2	75	1.42 ± 0.2
GGRAC8-150					150	0.71 ± 0.2
GGRAC8-250					250	0.50 ± 0.2
GGRAC10-75	12.7 ± 0.3	10	2.65 ± 0.5	9.5 ± 0.2	75	1.42 ± 0.2
GGRAC10-150					150	0.71 ± 0.2
GGRAC10-250					250	0.50 ± 0.2

**Table 5:** Testing results.

Sample Label	Peak Load (KN)	Axial Deflection at Peak Load (mm)	Ultimate Axial Deflections (mm)	Ductility Index
GGRAC6-75	1652.8	3.24	8.49	1.78 ± 0.15
GGRAC6-150	1520.7	3.11	8.11	1.65 ± 0.15
GGRAC6-250	1440.9	3.43	9.28	1.72 ± 0.15
GGRAC8-75	1777.3	3.25	8.79	1.93 ± 0.15
GGRAC8-150	1712.3	2.79	7.45	1.55 ± 0.15
GGRAC8-250	1571.1	3.36	7.23	1.43 ± 0.15
GGRAC10-75	1713.8	3.63	8.87	1.83 ± 0.15
GGRAC10-150	1587.4	3.56	7.13	1.51 ± 0.15
GGRAC10-250	1488.1	2.92	5.74	1.27 ± 0.15

**Table 6:** Statistical indices of developed database (COV represents the coefficient of variance and St. Dev represents the standard deviation).

Parameter	B (mm)	D (mm)	H (mm)	Ag (mm^2^)	fc’ (MPa)	$f_{u}$ (MPa)	$E_{f}$ (GPa)	$\varepsilon_{u}$ (%)	$\rho_{1}$ (%)	$\rho_{t}$ (%)	A_f_ (mm^2^)	$P_{n}$ (kN)
Minimum	150	150	150	17,662	20.0	406	23.4	0.97	0.55	0.01	212.53	114
Maximum	610	305	610	372,100	70.2	1680	141	2.42	5.3	5.3	4051.60	15,235
Mean	249	258	272	66,289	36.2	1010	56.7	1.78	2.09	1.38	1214.58	1814
St. Dev	114	54	114	53,039	12.6	339	25.1	0.39	1.06	1.06	764.62	1877
COV	0.46	0.21	0.43	0.81	0.35	0.34	0.45	0.22	0.51	0.77	0.63	1.04

## Data Availability

Data used in this work has been provided as a supplementary material.

## Supplementary Materials

The following are available online at https://www.mdpi.com/article/10.3390/polym13091508/s1. Table S1: Constructed Experimental Database.
