# Supplementary material for: On the Structural Performance of Recycled Aggregate Concrete Columns with Glass Fiber-Reinforced Composite Bars and Hoops
Source: Polymers (Basel). 2021 May 7;13(9):1508. doi: 10.3390/polym13091508 (PMC8125636; doi:10.3390/polym13091508)
Supplement: Supplementary file 1 [file polymers-13-01508-s001.zip › polymers-1194914-supplementary.pdf]

Table S1. Constructed experimetal database (GS = GFRP spirals, GH = GFRP hoops, SS = steel spirals, SH = steel hoops, N = no lateral reinforcement, AS = axial strength).

Sr. No .	Research study	B	H	D	f_c'	f_u	E_f	ε_u	Longitudinal bars		Transverse confinement		AS
		(mm)	(mm)	(mm)	(MPa)	(MPa)	(GPa)	(%)	Bars	ρ_l (%)	Type	ρ_t (%)	(kN)
1	Afifi et al. [1]	-	-	300	20	934	55.4	1.56	8 No. 5	2.2	GS	1	2920
2	Afifi et al. [1]	-	-	300	20	934	55.4	1.56	4 No. 5	1.1	GS	1	2826
3	Afifi et al. [1]	-	-	300	20	934	55.4	1.56	12 No. 5	3.2	GS	1	2998
4	Afifi et al. [1]	-	-	300	20	934	55.4	1.56	8 No. 5	2.2	GS	0.45	2857
5	Afifi et al. [1]	-	-	300	20	934	55.4	1.56	8 No. 5	2.2	GS	1.87	3019
6	Afifi et al. [1]	-	-	300	20	934	55.4	1.56	8 No. 5	2.2	GS	2.07	2964
7	Afifi et al. [1]	-	-	300	20	934	55.4	1.56	8 No. 5	2.2	GS	0.69	2804
8	Afifi et al. [1]	-	-	300	20	934	55.4	1.56	8 No. 5	2.2	GS	1.03	2951
9	Afifi et al. [1]	-	-	300	20	934	55.4	1.56	8 No. 5	2.2	GS	1.03	2865
10	Afifi et al. [2]	-	-	301	21	934	55.4	1.56	8 No. 5	2.2	GS	1.5	2840
11	Afifi et al. [2]	-	-	302	22	934	55.4	1.56	8 No. 5	2.2	GS	1.5	2871
12	Afifi et al. [2]	-	-	303	23	934	55.4	1.56	8 No. 5	2.2	GS	1.5	2935
13	AlAjarmeh et al. [3]	-	-	250	31.8	1237	60	2.1	6 No. 5	2.41	GS	1.49	1588
14	AlAjarmeh et al. [3]	-	-	250	31.8	1237	60	2.1	6 No. 5	2.47	GS	1.56	1408
15	AlAjarmeh et al. [3]	-	-	250	31.8	1237	60	2.1	6 No. 5	2.59	GS	1.69	1559
16	AlAjarmeh et al. [3]	-	-	250	31.8	1237	60	2.1	6 No. 5	2.78	GS	1.92	1411
17	AlAjarmeh et al. [4]	-	-	251	25	1281	61.3	2.1	6 No. 4	1.78	GS	1.57	1035.3
18	AlAjarmeh et al. [4]	-	-	252	25	1237	60.5	2.1	6 No. 5	2.79	GS	1.57	1109.2
19	AlAjarmeh et al. [4]	-	-	253	25	1270	60.5	2.1	6 No. 6	4	GS	1.57	1247.9
20	AlAjarmeh et al. [4]	-	-	254	25	1237	60.5	2.1	4 No. 5	1.86	GS	1.57	983.3
21	AlAjarmeh et al. [4]	-	-	255	25	1237	60.5	2.1	8 No. 5	3.72	GS	1.57	1406.1
22	AlAjarmeh et al. [4]	-	-	256	25	1281	61.3	2.1	9 No. 4	2.67	GS	1.57	1204.2
23	Alsayed et al. [5]	250	450	-	39	800	40	1.5	6 No. 5	1	SH	0.15	3285
24	Alsayed et al. [5]	250	450	-	39	800	40	1.5	6 No. 5	1	SH	0.15	3285
25	Alsayed et al. [5]	250	450	-	39	800	40	1.5	6 No. 5	1	SH	0.15	3285
26	Alsayed et al. [5]	250	450	-	38.5	800	40	1.5	6 No. 5	1	GH	0.18	3301
27	Alsayed et al. [5]	250	450	-	38.5	800	40	1.5	6 No. 5	1	GH	0.18	3301
28	Alsayed et al. [5]	250	450	-	38.5	800	40	1.5	6 No. 5	1	GH	0.18	3301
29	De Luca et al. [6]	610	610	-	43.7	608	44.2	1.38	8 No. 8	1	GH	0.63	15235
30	De Luca et al. [6]	610	610	-	40.6	712	44.4	1.6	8 No 8	1	GH	0.63	12949
31	De Luca et al. [6]	610	610	-	36.1	608	44.2	1.38	8 No. 8	1	GH	2.5	11926
32	De Luca et al. [6]	610	610	-	32.8	712	44.4	1.6	8 No 8	1	GH	2.5	10751
33	Dong et al. [7]	-	-	215	40	930	59	1.6	3 No. 3	0.55	GS	0.94	1018
34	Dong et al. [7]	-	-	215	40	930	59	1.6	4 No. 3	0.73	GS	0.94	1179
35	Dong et al. [7]	-	-	215	40	930	59	1.6	5 No. 3	0.92	GS	0.94	1288
36	Dong et al. [7]	-	-	215	40	930	59	1.6	6 No. 3	1.1	GS	0.94	1381
37	Dong et al. [7]	-	-	215	40	930	59	1.6	4 No. 3	0.73	GS	2.75	1459
38	Dong et al. [7]	-	-	215	40	930	59	1.6	4 No. 3	0.73	GS	2.75	1037

39	Dong et al. [7]	-	-	215	40	880	59	1.6	4 No. 3	0.73	GS	2.75	523
40	Dong et al. [7]	-	-	215	37	880	59	1.6	4 No. 3	0.73	GS	2.75	318
41	Dong et al. [7]	-	-	215	37	880	59	1.6	5 No. 3	0.73	GS	1.39	1290
42	Dong et al. [7]	-	-	215	37	880	59	1.6	6 No. 3	0.73	GS	1.39	944
43	Dong et al. [7]	-	-	215	37	880	59	1.6	7 No. 3	0.73	GS	1.39	527
44	Dong et al. [7]	-	-	215	37	880	59	1.6	8 No. 3	0.73	GS	1.39	296
45	Elchalakani and Ma [8]	160	260	-	32.8	1200	50	2.4	6 No. 4	1.8	GH	0.5	1367
46	Elchalakani and Ma [8]	160	260	-	32.8	1200	50	2.4	6 No. 4	1.8	GH	0.5	880
47	Elchalakani and Ma [8]	160	260	-	32.8	1200	50	2.4	6 No. 4	1.8	GH	0.5	584
48	Elchalakani and Ma [8]	160	260	-	32.8	1200	50	2.4	6 No. 4	1.8	GH	1	1449
49	Elchalakani and Ma [8]	160	260	-	32.8	1200	50	2.4	6 No. 4	1.8	GH	1	917
50	Elchalakani and Ma [8]	160	260	-	32.8	1200	50	2.4	6 No. 4	1.8	GH	1	788
51	Elchalakani and Ma [8]	160	260	-	32.8	1200	50	2.4	6 No. 4	1.8	GH	0.3	1402
52	Elchalakani et al. [9]	160	260	-	32.8	930	59	1.7	6 No. 4	1.8	GH	0.3	1402
53	Elchalakani et al. [9]	160	260	-	32.8	930	59	1.7	6 No. 4	1.8	GH	0.5	1367
54	Elchalakani et al. [9]	160	260	-	32.8	930	59	1.7	6 No. 4	1.8	GH	1	1449
55	Elchalakani et al. [9]	160	260	-	32.8	930	59	1.7	6 No. 4	1.8	GH	0.5	880
56	Elchalakani et al. [9]	160	260	-	32.8	930	59	1.7	6 No. 4	1.8	GH	1	917
57	Elchalakani et al. [9]	160	260	-	32.8	930	59	1.7	6 No. 4	1.8	GH	1	788
58	Elchalakani et al. [9]	160	260	-	32.8	930	59	1.7	6 No. 4	1.8	GH	0.5	584
59	Elchalakani et al. [9]	160	260	-	32.8	930	59	1.7	6 No. 4	1.8	GH	0.3	1041
60	Elchalakani et al. [9]	160	260	-	32.8	930	59	1.7	6 No. 4	1.8	GH	0.5	1194
61	Elchalakani et al. [9]	160	260	-	32.8	930	59	1.7	6 No. 4	1.8	GH	1	1357
62	Elchalakani et al. [9]	160	260	-	32.8	930	59	1.7	6 No. 4	1.8	GH	0.5	657
63	Elchalakani et al. [9]	160	260	-	32.8	930	59	1.7	6 No. 4	1.8	GH	1	804
64	Elchalakani et al. [9]	160	160	-	32.8	930	59	1.7	6 No. 4	1.8	GH	0.5	353
65	Elchalakani et al. [9]	160	160	-	32.8	930	59	1.7	6 No. 4	1.8	GH	1	454
66	Elchalakani et al. [9]	160	160	-	32.8	930	59	1.7	6 No. 4	1.8	GH	0.5	234
67	Elchalakani et al. [9]	160	160	-	32.8	930	59	1.7	6 No. 4	1.8	GH	1	244
68	Guerin et al. [10]	405	405	-	25.3	600	40	1.5	6 No. 6	1	GH	0.66	4587
69	Guerin et al. [10]	405	405	-	25.3	600	40	1.5	6 No. 6	1	GH	0.66	3433
70	Guerin et al. [10]	405	405	-	25.3	600	40	1.5	6 No. 6	1	GH	0.66	1591
71	Guerin et al. [10]	405	405	-	25.3	600	40	1.5	6 No. 6	1	GH	0.66	645
72	Guerin et al. [10]	405	405	-	25.3	600	40	1.5	6 No. 6	1	GH	0.66	4616
73	Guerin et al. [10]	405	405	-	25.3	600	40	1.5	6 No. 6	1	GH	0.66	3405
74	Guerin et al. [10]	405	405	-	25.3	600	40	1.5	6 No. 6	1	GH	0.66	1576
75	Guerin et al. [10]	405	405	-	25.3	600	40	1.5	6 No. 6	1	GH	0.66	636
76	Guerin et al. [11]	405	405	-	25.3	600	40	1.5	8 No. 6	1.4	GH	0.84	5028
77	Guerin et al. [11]	405	405	-	25.3	600	40	1.5	8 No. 6	1.4	GH	0.84	3627
78	Guerin et al. [11]	405	405	-	25.3	600	40	1.5	8 No. 6	1.4	GH	0.84	2035
79	Guerin et al. [11]	405	405	-	25.3	600	40	1.5	8 No. 6	1.4	GH	0.84	914
80	Guerin et al. [11]	405	405	-	25.3	600	40	1.5	8 No. 8	2.5	GH	0.63	5294

81	Guerin et al. [11]	405	405	-	25.3	600	40	1.5	8 No. 8	2.5	GH	0.63	3790
82	Guerin et al. [11]	405	405	-	25.3	600	40	1.5	8 No. 8	2.5	GH	0.63	2110
83	Guerin et al. [11]	405	405	-	25.3	600	40	1.5	8 No. 8	2.5	GH	0.63	1008
84	Hadhood et al. [12]	-	-	305	35	1680	141	1.19	8 No. 5	2.2	GH	2.68	2564
85	Hadhood et al. [12]	-	-	305	35	1680	141	1.19	8 No. 5	2.2	GH	2.68	2060
86	Hadhood et al. [12]	-	-	305	35	1680	141	1.19	8 No. 5	2.2	GH	2.68	1511
87	Hadhood et al. [12]	-	-	305	35	1680	141	1.19	8 No. 5	2.2	GH	2.68	776
88	Hadhood et al. [12]	-	-	305	35	1680	141	1.19	8 No. 5	2.2	GH	2.68	366
89	Hadhood et al. [12]	-	-	305	35	1680	141	1.19	8 No. 5	2.2	GS	1	2608
90	Hadhood et al. [12]	-	-	305	35	1680	141	1.19	8 No. 5	2.2	GS	1	2134
91	Hadhood et al. [12]	-	-	305	35	1680	141	1.19	8 No. 5	2.2	GS	1	1513
92	Hadhood et al. [12]	-	-	305	35	1680	141	1.19	8 No. 5	2.2	GS	1	745
93	Hadhood et al. [12]	-	-	305	35	1680	141	1.19	8 No. 5	2.2	GS	1	654
94	Hadhood et al. [12]	-	-	305	35	1680	141	1.19	12 No. 5	3.3	GS	1	2670
95	Hadhood et al. [12]	-	-	305	35	1680	141	1.19	12 No. 5	3.3	GS	1	2123
96	Hadhood et al. [12]	-	-	305	35	1680	141	1.19	12 No. 5	3.3	GS	1	1527
97	Hadhood et al. [12]	-	-	305	35	1680	141	1.19	12 No. 5	3.3	GS	1	852
98	Hadhood et al. [12]	-	-	305	35	1680	141	1.19	12 No. 5	3.3	GS	1	378
99	Hadhood et al. [13]	-	-	305	35	1680	141	1.19	8 No. 5	2.2	GS	1.8	2652
100	Hadhood et al. [13]	-	-	305	35	1680	141	1.19	8 No. 5	2.2	GS	1.8	2086
101	Hadhood et al. [13]	-	-	305	35	1680	141	1.19	8 No. 5	2.2	GS	1.8	1483
102	Hadhood et al. [13]	-	-	305	35	1680	141	1.19	8 No. 5	2.2	GS	1.8	747
103	Hadhood et al. [13]	-	-	305	35	1680	141	1.19	8 No. 5	2.2	GS	1.8	655
104	Hadhood et al. [13]	-	-	305	70.2	1289	54.9	2.3	8 No. 5	2.2	GS	1.1	4709
105	Hadhood et al. [13]	-	-	305	70.2	1289	54.9	2.3	8 No. 5	2.2	GS	1.1	3309
106	Hadhood et al. [13]	-	-	305	70.2	1289	54.9	2.3	8 No. 5	2.2	GS	1.1	2380
107	Hadhood et al. [13]	-	-	305	70.2	1289	54.9	2.3	8 No. 5	2.2	GS	1.1	1112
108	Hadhood et al. [13]	-	-	305	70.2	1289	54.9	2.3	8 No. 5	2.2	GS	1.1	797
109	Hadhood et al. [13]	-	-	305	70.2	1289	54.9	2.3	8 No. 5	2.2	GH	1.1	4689
110	Hadhood et al. [13]	-	-	305	70.2	1289	54.9	2.3	8 No. 5	2.2	GH	1.1	3299
111	Hadhood et al. [13]	-	-	305	70.2	1289	54.9	2.3	8 No. 5	2.2	GH	1.1	2435
112	Hadhood et al. [13]	-	-	305	70.2	1289	54.9	2.3	8 No. 5	2.2	GH	1.1	1054
113	Hadhood et al. [13]	-	-	305	70.2	1289	54.9	2.3	8 No. 5	2.2	GH	1.1	838
114	Hadhood et al. [13]	-	-	305	70.2	1289	54.9	2.3	12 No. 5	3.2	GS	1.1	4716
115	Hadhood et al. [13]	-	-	305	70.2	1289	54.9	2.3	12 No. 5	3.2	GS	1.1	3380
116	Hadhood et al. [13]	-	-	305	70.2	1289	54.9	2.3	12 No. 5	3.2	GS	1.1	2339
117	Hadhood et al. [13]	-	-	305	70.2	1289	54.9	2.3	12 No. 5	3.2	GS	1.1	1135
118	Hadhood et al. [13]	-	-	305	70.2	1289	54.9	2.3	12 No. 5	3.2	GS	1.1	713
119	Hadhood et al. [13]	-	-	305	70.2	1289	54.9	2.3	8 No. 5	2.2	GS	1.1	5120
120	Hadhood et al. [13]	-	-	305	70.2	1289	54.9	2.3	8 No. 5	2.2	GS	1.1	3671
121	Hadhood et al. [13]	-	-	305	70.2	1289	54.9	2.3	8 No. 5	2.2	GS	1.1	2538
122	Hadhood et al. [13]	-	-	305	70.2	1289	54.9	2.3	8 No. 5	2.2	GS	1.1	1392

123	Hadhood et al. [13]	-	-	305	70.2	1289	54.9	2.3	8 No. 5	2.2	GS	1.1	611
124	Hadhood et al. [13]	-	-	305	70.2	1289	54.9	2.3	8 No. 5	2.2	GS	1.7	4680
125	Hadhood et al. [13]	-	-	305	70.2	1289	54.9	2.3	8 No. 5	2.2	GS	1.7	3341
126	Hadhood et al. [13]	-	-	305	70.2	1289	54.9	2.3	8 No. 5	2.2	GS	1.7	2460
127	Hadhood et al. [13]	-	-	305	70.2	1289	54.9	2.3	8 No. 5	2.2	GS	1.7	1061
128	Hadhood et al. [13]	-	-	305	70.2	1289	54.9	2.3	8 No. 5	2.2	GS	1.7	682
129	Hadhood et al. [13]	-	-	305	35	1289	54.9	2.3	8 No. 5	2.2	GS	1.1	2608
130	Hadhood et al. [13]	-	-	305	35	1289	54.9	2.3	8 No. 5	2.2	GS	1.1	2134
131	Hadhood et al. [13]	-	-	305	35	1289	54.9	2.3	8 No. 5	2.2	GS	1.1	1512
132	Hadhood et al. [13]	-	-	305	35	1289	54.9	2.3	8 No. 5	2.2	GS	1.1	745
133	Hadhood et al. [13]	-	-	305	35	1289	54.9	2.3	8 No. 5	2.2	GS	1.1	354
134	Hadhood et al. [13]	-	-	305	35	1289	54.9	2.3	8 No. 5	2.2	GS	1.1	3090
135	Hadhood et al. [13]	-	-	305	35	1289	54.9	2.3	8 No. 5	2.2	GS	1.1	2342
136	Hadhood et al. [13]	-	-	305	35	1289	54.9	2.3	8 No. 5	2.2	GS	1.1	1746
137	Hadhood et al. [13]	-	-	305	35	1289	54.9	2.3	8 No. 5	2.2	GS	1.1	995
138	Hadhood et al. [13]	-	-	305	35	1289	54.9	2.3	8 No. 5	2.2	GS	1.1	529
139	Hadhood et al. [13]	-	-	305	35	1289	54.9	2.3	8 No. 5	2.2	GS	1.1	2652
140	Hadhood et al. [13]	-	-	305	35	1289	54.9	2.3	8 No. 5	2.2	GS	1.1	2086
141	Hadhood et al. [13]	-	-	305	35	1289	54.9	2.3	8 No. 5	2.2	GS	1.1	1483
142	Hadhood et al. [13]	-	-	305	35	1289	54.9	2.3	8 No. 5	2.2	GS	1.1	747
143	Hadhood et al. [13]	-	-	305	35	1289	54.9	2.3	8 No. 5	2.2	GS	1.1	355
144	Hadi et al. [14]	-	-	205	37	1200	50	2.4	6 No. 4	1.6	GS	2.1	1220
145	Hadi et al. [14]	-	-	205	37	1200	50	2.4	6 No. 4	1.6	GS	2.1	781
146	Hadi et al. [14]	-	-	205	37	1200	50	2.4	6 No. 4	1.6	GS	2.1	494
147	Hadi et al. [14]	-	-	205	37	1200	50	2.4	6 No. 4	1.6	GS	4.2	1309
148	Hadi et al. [14]	-	-	205	37	1200	50	2.4	6 No. 4	1.6	GS	4.2	767
149	Hadi et al. [14]	-	-	205	37	1200	50	2.4	6 No. 4	1.6	GS	4.2	479
150	Hadi and Youssef [15]	210	210	-	29.3	1641	67.9	2.41	4 No. 4	1	GH	2.74	1285
151	Hadi and Youssef [15]	210	210	-	29.3	1641	67.9	2.41	4 No. 4	1	GH	2.74	803
152	Hadi and Youssef [15]	210	210	-	29.3	1641	67.9	2.41	4 No. 4	1	GH	2.74	615
153	Hassan et al. [16]	-	-	150	40	800	30	0.97	6 No. 3	2.1	SS	1.7	426.59
154	Hassan et al. [16]	-	-	150	40	800	30	1.35	6 No. 3	2.1	SS	1.7	411.88
155	Hassan et al. [16]	-	-	150	40	800	30	1.57	6 No. 3	2.1	SS	1.7	387.36
156	Hassan et al. [16]	-	-	150	40	800	30	1.4	6 No. 3	2.1	SS	3.4	529.56
157	Hassan et al. [16]	-	-	150	40	800	30	1.7	6 No. 3	2.1	SS	3.4	490.33
158	Hassan et al. [16]	-	-	150	40	800	30	1.9	6 No. 3	2.1	SS	3.4	460.91
159	Hassan et al. [16]	-	-	150	40	800	30	1.28	6 No. 3	2.1	GH	1.7	490.33
160	Hassan et al. [16]	-	-	150	40	800	30	1.5	6 No. 3	2.1	GH	1.7	460.91
161	Hassan et al. [16]	-	-	150	40	800	30	1.7	6 No. 3	2.1	GH	1.7	430.4
162	Karim et al. [17]	-	-	205	37	1600	66	2.42	6 No. 4	4.72	GS	1.91	1425
163	Karim et al. [17]	-	-	205	37	1600	66	2.42	6 No. 4	4.72	GS	3.82	2041
164	Karim et al. [12]	-	-	206	37	1600	66	2.42	6 No. 4	4.72	GS	1.91	1425

165	Karim et al. [12]	-	-	207	37	1600	66	2.42	6 No. 4	4.72	GS	1.91	781
166	Karim et al. [12]	-	-	208	37	1600	66	2.42	6 No. 4	4.72	GS	1.91	494
167	Karim et al. [12]	-	-	209	37	1600	66	2.42	6 No. 4	4.72	GS	3.82	2041
168	Karim et al. [12]	-	-	210	37	1600	66	2.42	6 No. 4	4.72	GS	3.82	767
169	Karim et al. [12]	-	-	211	37	1600	66	2.42	6 No. 4	4.72	GS	3.82	479
170	Karim et al. [12]	-	-	212	37	1600	66	2.42	6 No. 4	4.72	GS	1.91	3068
171	Karim et al. [12]	-	-	213	37	1600	66	2.42	6 No. 4	4.72	GS	1.91	1450
172	Karim et al. [12]	-	-	214	37	1600	66	2.42	6 No. 4	4.72	GS	1.91	805
173	Khan et al. [18]	-	-	206	37	1395	56	1.5	6 No. 5	3.57	GH	-	2812
174	Khan et al. [18]	-	-	206	37	1395	56	1.5	6 No. 5	3.57	GH	-	1487
175	Khan et al. [18]	-	-	206	37	1395	56	1.5	6 No. 5	3.57	GH	-	910
176	Khorramian &Sadeghian [19]	150	150	-	37	629	38.7	1.62	6 No. 5	5.3	N	-	775
177	Khorramian & Sadeghian [19]	150	150	-	37	629	38.7	1.62	6 No. 5	5.3	N	-	775
178	Khorramian &Sadeghian [19]	150	150	-	37	629	38.7	1.62	6 No. 5	5.3	N	-	693
179	Khorramian &Sadeghian [19]	150	150	-	37	629	38.7	1.62	6 No. 5	5.3	N	-	693
180	Khorramian &Sadeghian [19]	150	150	-	37	629	38.7	1.62	6 No. 5	5.3	N	-	693
181	Khorramian &Sadeghian [19]	150	150	-	37	629	38.7	1.62	6 No. 5	5.3	N	-	578
182	Khorramian &Sadeghian [19]	150	150	-	37	629	38.7	1.62	6 No. 5	5.3	N	-	578
183	Khorramian &Sadeghian [19]	150	150	-	37	629	38.7	1.62	6 No. 5	5.3	N	-	354
184	Khorramian &Sadeghian [19]	150	150	-	37	629	38.7	1.62	6 No. 5	5.3	N	-	354
185	Maranan et al. [20]	-	-	250	34.42	1184	62.6	1.89	6 No. 5	2.43	GH	3.13	1772
186	Maranan et al. [20]	-	-	250	34.42	1184	62.6	1.89	6 No. 5	2.43	GH	3.13	1791
187	Maranan et al. [20]	-	-	250	34.42	1184	62.6	1.89	6 No. 5	2.43	GH	1.57	1981
188	Maranan et al. [20]	-	-	250	34.42	1184	62.6	1.89	6 No. 5	2.43	GH	0.78	1988
189	Maranan et al. [20]	-	-	250	34.42	1184	62.6	1.89	6 No. 5	2.43	GS	3.13	1838
190	Maranan et al. [20]	-	-	250	34.42	1184	62.6	1.89	6 No. 5	2.43	GS	1.57	2063
191	Maranan et al. [20]	-	-	250	34.42	1184	62.6	1.89	6 No. 5	2.43	GH	1.57	1624
192	Maranan et al. [20]	-	-	250	34.42	1184	62.6	1.89	6 No. 5	2.43	GS	1.57	1208
193	Mohamed et al. [21]	-	-	300	42.9	934	55.4	1.56	8 No. 5	2.2	GH	2.23	2840
194	Mohamed et al. [21]	-	-	300	42.9	934	55.4	1.56	8 No. 5	2.2	GH	2.68	2871
195	Mohamed et al. [21]	-	-	300	42.9	934	55.4	1.56	8 No. 5	2.2	GH	3.14	2935
196	Pantelides et al. [22]	-	-	254	36	740	43.3	1.71	4 No. 5	1.6	GS	0.75	1975
197	Pantelides et al. [22]	-	-	254	36	740	43.3	1.71	4 No. 5	1.6	GS	0.75	1788
198	Prachasaree et al. [23]	150	150	-	20.8	735	50	1.5	4 No. 3	1.4	SS	0.01	370
199	Prachasaree et al. [23]	150	150	-	20.8	735	50	1.5	4 No. 3	1.4	SS	0.01	370
200	Prachasaree et al. [23]	150	150	-	20.8	735	50	1.5	4 No. 3	1.4	SS	0.01	370
201	Prachasaree et al. [23]	150	150	-	20.8	735	50	1.5	4 No. 3	1.4	SS	0.02	365
202	Prachasaree et al. [23]	150	150	-	20.8	735	50	1.5	4 No. 3	1.4	SS	0.02	365
203	Prachasaree et al. [23]	150	150	-	20.8	735	50	1.5	4 No. 3	1.4	SS	0.02	365
204	Prachasaree et al. [23]	-	-	150	20.8	735	50	1.5	4 No. 3	1.9	SS	0.01	345
205	Prachasaree et al. [23]	-	-	150	20.8	735	50	1.5	4 No. 3	1.9	SS	0.01	345
206	Prachasaree et al. [23]	-	-	150	20.8	735	50	1.5	4 No. 3	1.9	SS	0.01	345

207	Prachasaree et al. [23]	-	-	150	20.8	735	50	1.5	4 No. 3	1.9	SS	0.02	315
208	Prachasaree et al. [23]	-	-	150	20.8	735	50	1.5	4 No. 3	1.9	SS	0.02	315
209	Prachasaree et al. [23]	-	-	150	20.8	735	50	1.5	4 No. 3	1.9	SS	0.02	315
210	Prachasaree et al. [23]	150	150	-	20.8	735	50	1.5	4 No. 3	1.4	SH	0.01	365
211	Prachasaree et al. [23]	150	150	-	20.8	735	50	1.5	4 No. 3	1.4	SH	0.01	365
212	Prachasaree et al. [23]	150	150	-	20.8	735	50	1.5	4 No. 3	1.4	SH	0.01	365
213	Prachasaree et al. [23]	150	150	-	20.8	735	50	1.5	4 No. 3	1.4	SH	0.02	370
214	Prachasaree et al. [23]	150	150	-	20.8	735	50	1.5	4 No. 3	1.4	SH	0.02	370
215	Prachasaree et al. [23]	150	150	-	20.8	735	50	1.5	4 No. 3	1.4	SH	0.02	370
216	Sankholkar et al. [24]	-	-	203	50	800	46.2	1.57	4 No. 5	2.5	GS	3.2	1353
217	Sankholkar et al. [24]	-	-	203	50	800	46.2	1.57	4 No. 5	2.5	GS	3.2	1285
218	Sankholkar et al. [24]	-	-	203	50	800	46.2	1.57	6 No. 5	3.7	GS	3.2	1623
219	Sankholkar et al. [24]	-	-	203	50	800	46.2	1.57	6 No. 5	3.7	GS	3.2	1570
220	Sun et al. [25]	150	150	-	23.51	1103	54.1	1.5	6 No. 3	1.04	SH	0.63	201
221	Sun et al. [25]	150	150	-	23.51	1103	54.1	1.5	6 No. 3	1.04	SH	0.63	174
222	Sun et al. [25]	150	150	-	23.51	1103	54.1	1.5	6 No. 3	1.04	SH	0.63	181
223	Sun et al. [25]	150	150	-	23.51	1103	54.1	1.5	6 No. 3	1.04	SH	0.63	291
224	Sun et al. [25]	150	150	-	23.51	1103	54.1	1.5	6 No. 3	1.04	SH	0.63	290
225	Sun et al. [25]	150	150	-	23.51	1103	54.1	1.5	6 No. 3	1.04	SH	0.63	347
226	Sun et al. [25]	150	150	-	23.51	1103	54.1	1.5	6 No. 3	1.04	SH	0.63	632
227	Sun et al. [25]	150	150	-	23.51	1103	54.1	1.5	6 No. 3	1.04	SH	0.63	677
228	Sun et al. [25]	150	150	-	23.51	1103	54.1	1.5	6 No. 3	1.04	SH	0.63	602
229	Tikka et al. [26]	150	150	-	25.7	630	40	1.5	4 No. 4	2.3	CS	0.33	401
230	Tikka et al. [26]	150	150	-	25.7	630	40	1.5	4 No. 4	2.3	CS	0.33	120
231	Tikka et al. [26]	150	150	-	25.7	630	40	1.5	6 No. 4	3.4	CS	0.33	215
232	Tikka et al. [26]	150	150	-	25.7	630	40	1.5	4 No. 4	2.3	CS	0.33	382
233	Tikka et al. [26]	150	150	-	25.7	630	40	1.5	4 No. 4	2.3	CS	0.33	129
234	Tikka et al. [26]	150	150	-	25.7	630	40	1.5	6 No. 4	3.4	CS	0.33	220
235	Tikka et al. [26]	150	150	-	25.7	630	40	1.5	6 No. 4	3.4	CS	0.33	116
236	Tobbi et al. [27]	350	350	-	32.6	728	47.6	1.53	8 No. 6	1.9	GH	2	3929
237	Tobbi et al. [27]	350	350	-	32.6	728	47.6	1.53	8 No. 6	1.9	GH	2	3991
238	Tobbi et al. [27]	350	350	-	32.6	728	47.6	1.53	9 No. 6	1.9	GH	1.7	4006
239	Tobbi et al. [27]	350	350	-	32.6	752	48.2	1.56	12 No. 5	1.9	GH	3.2	3938
240	Tobbi et al. [27]	350	350	-	32.6	751	48.2	1.56	12 No. 5	1.9	GH	4.8	4067
241	Tobbi et al. [27]	350	350	-	36.4	750	48.2	1.56	8 No. 6	1.9	GH	2.55	4297
242	Tobbi et al. [27]	350	350	-	36.4	749	48.2	1.56	12 No. 5	1.9	GH	3.41	4615
243	Tobbi et al. [27]	350	350	-	36.4	748	48.2	1.56	4 No. 4 &5	1	GH	2.55	4212
244	Tobbi et al. [27]	350	350	-	36.4	747	48.2	1.56	8 No. 4	0.8	GH	2.55	3900
245	Tu et al. [28]	200	200	-	32.1	660	44.25	1.52	4 No. 4	1.1	GH	5.3	970.9
246	Tu et al. [28]	200	200	-	32.1	660	44.25	1.52	4 No. 4	1.1	GH	3.1	951.6
247	Tu et al. [28]	200	200	-	32.1	660	44.25	1.52	4 No. 4	1.1	GH	2	937.7
248	Tu et al. [28]	200	200	-	32.1	735	46	1.6	4 No. 3	0.8	GH	3.1	936.8

249	Tu et al. [28]	200	200	-	32.1	660	44.25	1.52	4 No. 4	1.5	GH	3.1	981.7
250	Tu et al. [28]	200	200	-	32.1	660	44.25	1.52	4 No. 4	1.1	GH	5.2	954

References

1. Afifi, M.Z.; Mohamed, H.M. and Benmokrane, B. Axial capacity of circular concrete columns reinforced with GFRP bars and spirals. *Journal of Composites for Construction*, **2014**, *18*(1), 04013017. https://doi.org/10.1061/(asce)cc.1943-5614.0000438
2. Afifi, M.Z.; Mohamed, H.M. and Benmokrane, B. Theoretical stress–strain model for circular concrete columns confined by GFRP spirals and hoops. *Engineering Structures*, **2015**, *102*, 202-213. https://doi.org/10.1016/j.engstruct.2015.08.020
3. AlAjarmeh, O.S.; Manalo, A.C.; Benmokrane, B.; Karunasena, W.; Mendis, P. and Nguyen, K.T.Q. Compressive behavior of axially loaded circular hollow concrete columns reinforced with GFRP bars and spirals. *Construction and Building Materials*, **2019**, *194*, 12-23. https://doi.org/10.1016/j.conbuildmat.2018.11.016
4. AlAjarmeh, O.S.; Manalo, A.C.; Benmokrane, B.; Karunasena, W. and Mendis, P. Axial performance of hollow concrete columns reinforced with GFRP composite bars with different reinforcement ratios. *Composite Structures*, **2019**, *213*, 153-164. https://doi.org/10.1016/j.compstruct.2019.01.096
5. Alsayed, S.H.; Al-Salloum, Y.A.; Almusallam, T.H. and Amjad, M.A.Concrete columns reinforced by glass fiber reinforced polymer rods. *Special Publication*, **1999**, *188*, 103-112.
6. De Luca, A.; Matta, F. and Nanni, A. Behavior of full-scale GFRP reinforced concrete columns under axial load. *ACI Structural Journal*, **2010**. *107*(5), 589-596. https://doi.org/10.14359/51663912
7. Dong, M.; Elchalakani, M.; Karrech, A.; Pham, T.M. and Yang, B. Glass fibre-reinforced polymer circular alkali-activated fly ash/slag concrete members under combined loading. *Engineering Structures*, **2019**, *199*, 109598. https://doi.org/10.1016/j.engstruct.2019.109598
8. Elchalakani, M. and Ma, G. Tests of glass fibre reinforced polymer rectangular concrete columns subjected to concentric and eccentric axial loading. *Engineering Structures*, **2017**, *151*, 93-104. https://doi.org/10.1016/j.engstruct.2017.08.023
9. Elchalakani, M.; Ma, G.; Aslani, F. and Duan, W. Design of GFRP-reinforced rectangular concrete columns under eccentric axial loading. *Magazine of Concrete Research*, **2017**, *69*(17), 865-877. https://doi.org/10.1680/jmacr.16.00437
10. Guérin, M.; Mohamed, H.M.; Benmokrane, B.; Nanni, A. and Shield, C.K. Eccentric Behavior of Full-Scale Reinforced Concrete Columns with Glass Fiber-Reinforced Polymer Bars and Ties. *ACI Structural Journal*, **2018**, *115*(2). https://doi.org/10.14359/51701107
11. Guérin, M.; Mohamed, H.M.; Benmokrane, B.; Shield, C.K. and Nanni, A. Effect of glass fiber-reinforced polymer reinforcement ratio on axial-flexural strength of reinforced concrete columns. *ACI Structural Journal*, **2018**, *115*(4), 1049-3. https://doi.org/10.14359/51701279
12. Hadhood, A.; Mohamed, H.M. and Benmokrane, B. Axial load–moment interaction diagram of circular concrete columns reinforced with CFRP bars and spirals: experimental and theoretical investigations. *Journal of Composites for Construction*, **2017**, *21*(2), 04016092. https://doi.org/10.1061/(asce)cc.1943-5614.0000748
13. Hadhood, A.; Mohamed, H.M. and Benmokrane, B. Assessing stress-block parameters in designing circular high-strength concrete members reinforced with FRP bars. *Journal of Structural Engineering*, **2018**, *144*(10), 04018182. https://doi.org/10.1061/(asce)st.1943-541x.0002173
14. Hadi, M.N.; Karim, H. and Sheikh, M.N. Experimental investigations on circular concrete columns reinforced with GFRP bars and helices under different loading conditions. *Journal of Composites for Construction*, **2016,** *20*(4), 04016009. https://doi.org/10.1061/(asce)cc.1943-5614.0000670

15. Hadi, M.N. and Youssef, J. Experimental investigation of GFRP-reinforced and GFRP-encased square concrete specimens under axial and eccentric load, and four-point bending test. *Journal of Composites for Construction*, **2016**, *20*(5), 04016020. https://doi.org/10.1061/(asce)cc.1943-5614.0000675
16. Hassan, A.; Khairallah, F.; Mamdouh, H. and Kamal, M. Structural behaviour of self-compacting concrete columns reinforced by steel and glass fibre-reinforced polymer rebars under eccentric loads. *Engineering Structures*, **2019**, *188*, 717-728. https://doi.org/10.1016/j.engstruct.2019.03.067
17. Karim, H.; Sheikh, M.N. and Hadi, M.N. Axial load-axial deformation behaviour of circular concrete columns reinforced with GFRP bars and helices. *Construction and Building Materials*, **2016**, *112*, 1147-1157. https://doi.org/10.1016/j.conbuildmat.2016.02.219
18. Khan, Q.S.; Sheikh, M.N. and Hadi, M.N. Axial-flexural interactions of GFRP-CFFT columns with and without reinforcing GFRP bars. *Journal of composites for construction*, **2017**, *21*(3), 04016109. https://doi.org/10.1061/(asce)cc.1943-5614.0000771
19. Khorramian, K. and Sadeghian, P. Experimental and analytical behavior of short concrete columns reinforced with GFRP bars under eccentric loading. *Engineering structures*, **2017**, *151*, 761-773. https://doi.org/10.1016/j.engstruct.2017.08.064
20. Maranan, G.B.; Manalo, A.C.; Benmokrane, B.; Karunasena, W. and Mendis, P. Behavior of concentrically loaded geopolymer-concrete circular columns reinforced longitudinally and transversely with GFRP bars. *Engineering Structures*, **2016**, *117*, 422-436. https://doi.org/10.1016/j.engstruct.2016.03.036
21. Mohamed, H.M.; Afifi, M.Z. and Benmokrane, B. Performance evaluation of concrete columns reinforced longitudinally with FRP bars and confined with FRP hoops and spirals under axial load. *Journal of Bridge Engineering*, **2014**, *19*(7), 04014020. https://doi.org/10.1061/(asce)be.1943-5592.0000590
22. Pantelides, C.P.; Gibbons, M.E. and Reaveley, L.D. Axial load behavior of concrete columns confined with GFRP spirals. *Journal of Composites for Construction*, **2013**, *17*(3), 305-313. https://doi.org/10.1061/(asce)cc.1943-5614.0000357
23. Prachasaree, W.; Piriyakootorn, S.; Sangsrijun, A. and Limkatanyu, S. Behavior and performance of GFRP reinforced concrete columns with various types of stirrups. *International Journal of Polymer Science*, **2015**, 2015. https://doi.org/10.1155/2015/237231
24. Sankholkar, P.P.; Pantelides, C.P. and Hales, T.A. Confinement model for concrete columns reinforced with GFRP spirals. *Journal of Composites for Construction*, **2018**, *22*(3), 04018007. https://doi.org/10.1061/(asce)cc.1943-5614.0000843
25. Sun, L.; Wei, M. and Zhang, N. Experimental study on the behavior of GFRP reinforced concrete columns under eccentric axial load. *Construction and Building Materials*, **2017**, *152*, 214-225. https://doi.org/10.1016/j.conbuildmat.2017.06.159
26. Tikka, T.; Francis, M. and Teng, B. Strength of concrete beam-columns reinforced with GFRP bars. in 2nd International Structures Specialty Conference, Winnipeg, MB, Canada. 2010.
27. Tobbi, H.; Farghaly, A.S. and Benmokrane, B. Concrete Columns Reinforced Longitudinally and Transversally with Glass Fiber-Reinforced Polymer Bars. *ACI Structural Journal*, **2012**, *109*(4). https://doi.org/10.14359/51683874
28. Tu, J.; Gao, K.; He, L. and Li, X. Experimental study on the axial compression performance of GFRP-reinforced concrete square columns. *Advances in Structural Engineering*, **2019**, *22*(7), 1554-1565. https://doi.org/10.1177/1369433218817988
